# Protein array processing software for automated semiquantitative analysis of serum antibody repertoires

**DOI:** 10.1063/5.0169421

**Published:** 2023-09-15

**Authors:** Ajeet Singh Yadav, Chin Hong Ooi, Hongjie An, Nam-Trung Nguyen, Gregor S. Kijanka

**Affiliations:** Queensland Micro-Nanotechnology Centre, Griffith University, Nathan, QLD 4111, Australia

## Abstract

Effective immunotherapies activate natural antitumor immune responses in patients undergoing treatment. The ability to monitor immune activation in response to immunotherapy is critical in measuring treatment efficacy over time and across patient cohorts. Protein arrays are systematically arranged, large collections of annotated proteins on planar surfaces, which can be used for the characterization of disease-specific and treatment-induced antibody repertoires in individuals undergoing immunotherapy. However, the absence of appropriate image analysis and data processing software presents a substantial hurdle, limiting the uptake of this approach in immunotherapy research. We developed a first, automated semiquantitative open-source software package for the analysis of widely used protein macroarrays. The software allows accurate single array and inter-array comparative studies through the tackling of intra-array inconsistencies arising from experimental disparities. The innovative and automated image analysis process includes adaptive positioning, background identification and subtraction, removal of null signals, robust statistical analysis, and protein pair validation. The normalized values allow a convenient semiquantitative data analysis of different samples or timepoints. Enabling accurate characterization of sample series to identify disease-specific immune profiles or their relative changes in response to treatment may serve as a diagnostic or predictive tool of disease.

## INTRODUCTION

Protein arrays are a well-established proteomic tool for the simultaneous analysis of thousands of interaction partners, generally manufactured in a microarray format through the immobilization of purified proteins on chemically modified microscope slides.^1^ An alternative to the microarray format are protein macroarrays produced by printing annotated libraries of *E. coli* clones, expressing recombinant human protein, on large 22 × 22 cm polyvinylidene fluoride (PVDF) membranes.^2,3^ Since their introduction in the early 2000s, the protein macroarrays have been frequently used with around 100 published studies in a wide range of applications from protein–protein,^4,5^ peptide–protein,^6,7^ enzyme–substrate,^8,9^ and post-translational modification interaction studies,^10,11^ to antibody specificity validation,^12,13^ antibody target discovery,^14,15^ antibody isotyping,^16–18^ and clinical autoantibody screening.^11,16,19^ The extensive usage of the protein macroarrays can be attributed to two innate characteristics unique to the platform: (i) *E.coli* expression clones are spotted directly on PVDF membranes, ensuring consistent protein concentrations intrinsic to each individual expression clone, thereby avoiding the need for cumbersome large-scale protein purification and characterization procedures essential for the generation of most protein microarray formats; and (ii) each individual colony spot on the macroarray comprises a single recombinant human protein and a collection of all *E.coli* proteins, thereby providing a natural bacterial protein blocking background consistent across the entire array and, hence, ensuring excellent experimental signal-to-noise ratios (SNRs).^20^

While users of protein microarrays may draw on dedicated commercial and open-source microarray analysis software packages,^21,22^ such packages are not suitable for protein macroarrays due to the large array size and associated image resolution characteristics.^23,24^ Protein macroarray analysis options are, thus, far limited to image data processing of subsections of the array^25,26^ or universal manufacturer-provided scanner software packages.^10^ More dedicated software packages such as VisualGrid (GPC Biotech)^19,27^ or Aida Image Analyser (Raytest)^5,28^ rely on either operator-based decision making or fixed spot diameter measurements. Both are not suitable for semiquantitative comparative studies. Here, we aim to develop an automated open-source software package dedicated to the semiquantitative analysis of protein macroarrays. The applications of the software range from non-clinical protein array binding studies to disease-associated immune antibody profiles with particular relevance to the comparative cohort or longitudinal clinical studies such as analyses of the response to immunotherapy treatment. We use MathWorks MATLAB version 2019b as the platform. The code is available for use in the supplementary material.

## EXPERIMENTAL PROCEDURES

### Protein macroarrays

HexSelect protein macroarrays (Engine GmbH, Germany) were applied as previously described.^19^ A human serum sample was selected randomly from a melanoma immunotherapy study approved by the Metro South Human Research Ethics Committee, Brisbane, Australia (HREC/16/QPAH/342); patient data, and clinical and treatment status were not taken into consideration for this current study. Briefly, protein macroarrays were incubated with the diluted serum (1:100) for 16 h. Mouse anti-human IgG antibody (GG-7, Sigma-Aldrich), alkaline phosphatase (AP)-conjugated goat anti-mouse IgG antibody (A1418, Sigma-Aldrich), and AttoPhos substrate (S1000; Promega) were used as detection reagents. The arrays were scanned using a GE Typhoon FLA 9000 Gel Imaging Scanner (GE Healthcare, Chicago, IL, USA).

### MATLAB programming

MATLAB code is written specifically for the HexSelect protein macroarrays imaged using a 16-bit scanner at a resolution of 10 pixels/mm. The user interface built into the code requires MATLAB version 2019b or later to work properly. We used uncompressed TIFF files converted from the scanner RAW file without any compression or noise removal for the best results. All images were checked to ensure that there was no pixel saturation; i.e., all pixel values were well within the linear dynamic range of 0 to 65 535. These macroarrays contain 57 600 “dots” that are grouped into 2304 (240 × 240) “cells.” Each cell consists of a 5 × 5 array of 25 dots, including the marker dot at the center and 12 pairs of clones in a specific configuration as previously shown.^19^ Each dot is represented by 9 × 9 pixels in the image.

## RESULTS AND DISCUSSION

### Retrieving a serum antibody repertoire

**FIG. 1.**
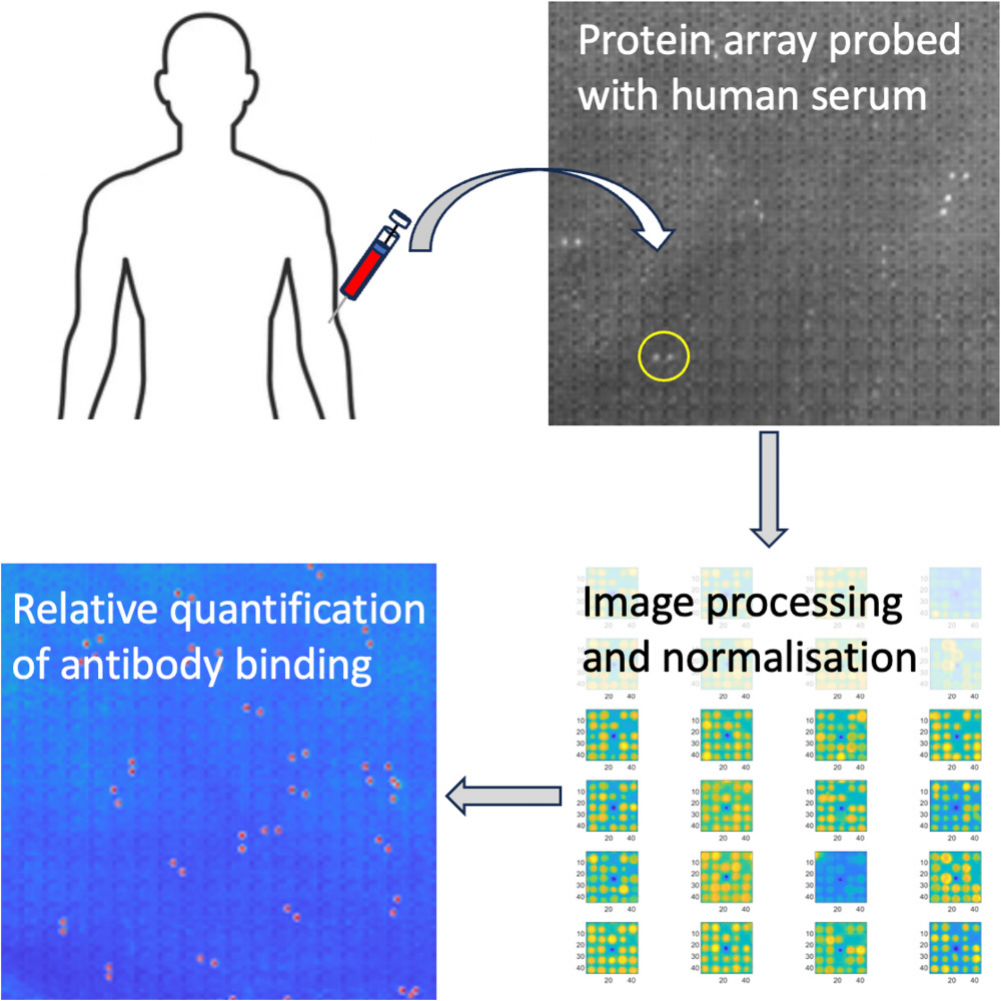
Semiquantitative analysis of antibody repertoires from human serum using protein macroarrays and specialized software for relative expression comparison between different patients and time points. An example of a positive signal represented by a pair of white dots is highlighted by a yellow circle.

A protein macroarray was incubated with human serum overnight to allow the binding of serum antibodies to the corresponding human proteins (Fig. 1). Antibody repertoires specific to each individual, their disease status, and treatment response can be next visualized using appropriate secondary antibodies.^18,19,29^ We have probed the array with an anti-human immunoglobulin G (IgG) secondary antibody pair to reveal the IgG antibody repertoire of the analyzed sample. The protein macroarray was scanned and showed a large number of positive signals typical for a human serum sample.^19,30^ The scanned image was next processed using the novel semiquantitative protein array analysis software.

### Image loading and aligning of the array

The protein macroarray TIFF image was loaded from the MATLAB folder via a user dialogue. The image may be mirrored depending on the side of the array that was scanned. The images used in this study were all of the same size of 2250 × 2250 pixels.

An accurate dot positioning is critical for large protein arrays as minute distortions present in the substrate accumulate throughout the entire array. Nevertheless, the large dimensions facilitate accurate physical placement in terms of orientation and, thus, substantially reduce positioning errors arising from rotation. Array scanners tend to sacrifice spatial resolution to improve the intensity resolution and signal-to-noise ratio (SNR) by using charge-coupled device (CCD) cameras with relatively large pixels. Therefore, positioning accuracy is paramount to reliable data.

Physical distortion of the array substrate is non-linear and heterogenous. As such, the ubiquitous three-point interpolation positioning method that assumes a perfect grid-like array structure fails to accurately determine dot locations in large arrays. To address this issue, we implement a two-stage positioning method that includes a four-point interpolation positioning method followed by a secondary adaptive position refinement aided by marker dots located at the center of each cell.

The coordinates of the marker dots at the four corners of the array are the only user inputs required by our method.

The four-point positioning method forms a quadrilateral with evenly interpolated spacing as an initial approximation to locate marker dots. As the dots were printed black with high contrast, the center of each marker dot is accurately determined via simple pixel intensity thresholding. Once determined, the pixel coordinates of the center of each marker dot form the center of each cell.

**FIG. 2.**
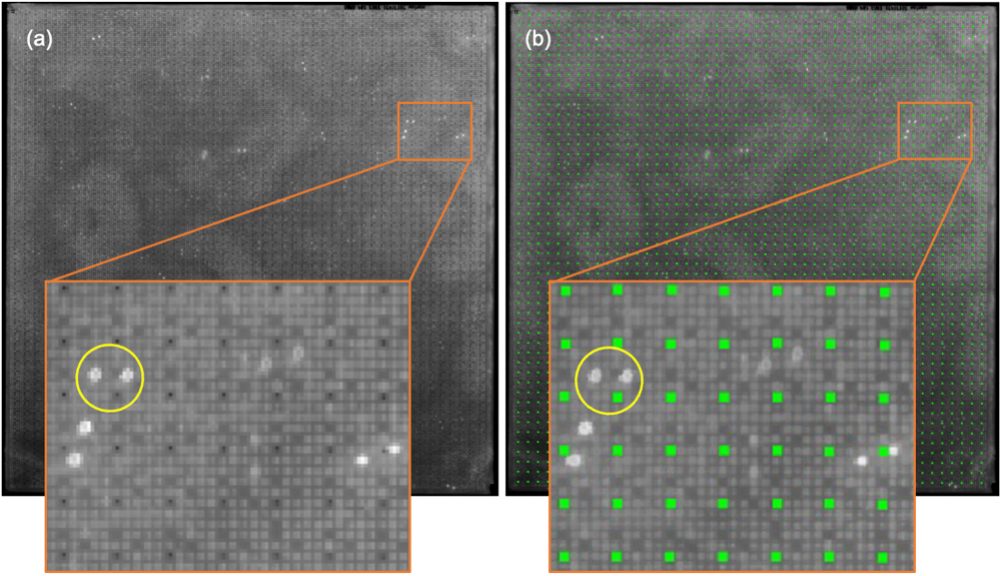
Determining the positions of the marker dots. (a) Unprocessed image of the protein macroarray: the heterogenous background is clearly visible. (b) Image overlaid with green marker positions matching the positions of printed black marker dots. Insets show a close-up view of the array with a positive signal represented by a pair of white dots highlighted by a yellow circle.

Figure 2 shows the positions of the marker dots acquired using this method.

### Reconstruction of array

**FIG. 3.**
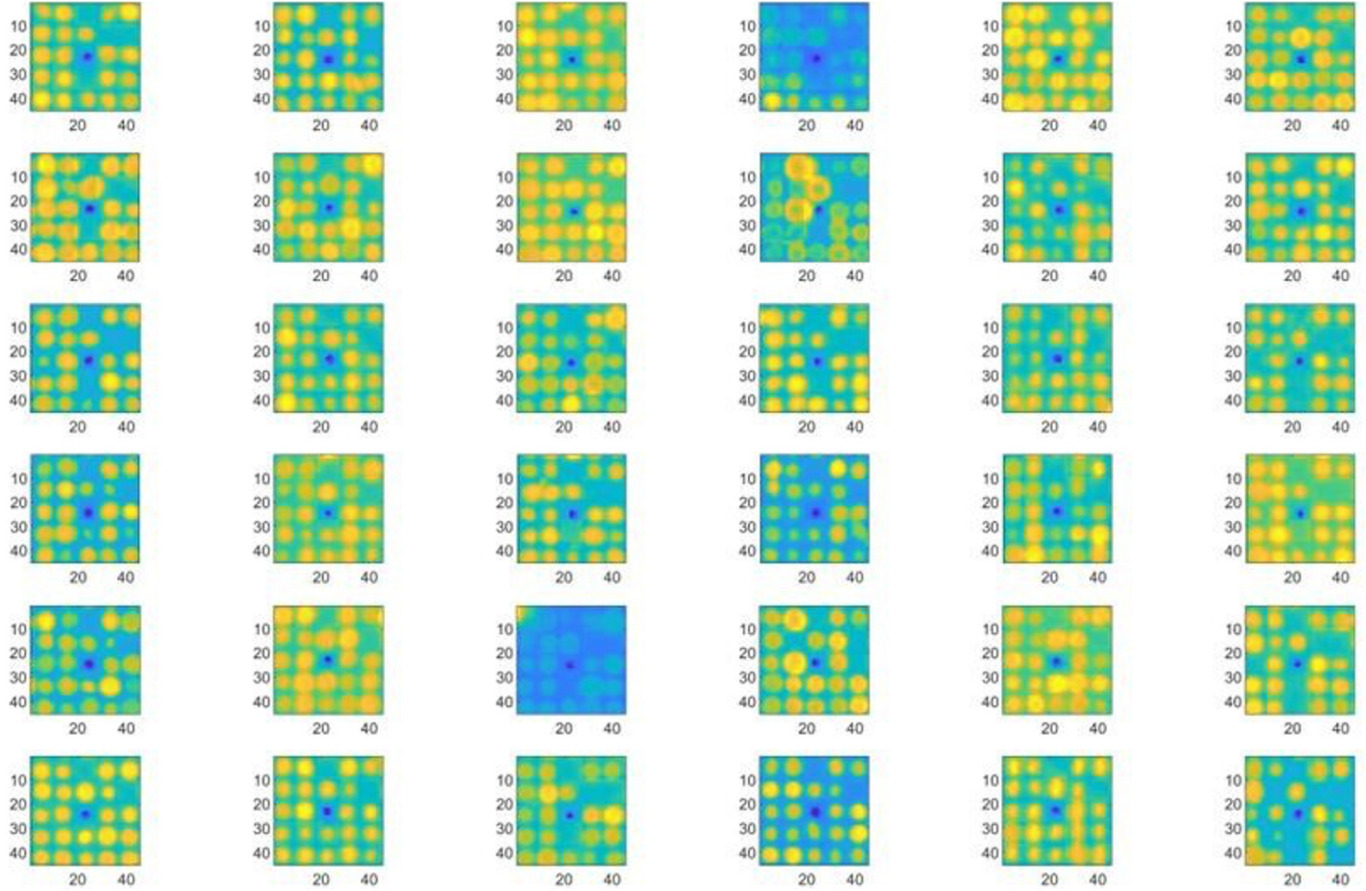
Sample cells to illustrate positioning accuracy throughout the entire array. The color is applied by MATLAB and normalized according to the pixel intensity. The marker dots are visible at the center of each cell in dark blue as they have the lowest pixel intensities. Numbers on the x and y axes of each cell represent the pixel count coordinates.

The array is next reconstructed such that the image analysis is only applied to regions of interest (2160 × 2160 pixels). Each cell is redefined by using the marker dot center as datum such that the cell area is ±22 pixels in the horizontal and vertical direction from the center pixel. To check the accuracy of the marker dot position and subsequent reconstruction, the code samples 6 × 6 cells that are evenly spaced across the entire array. A sample result is shown in Fig. 3.

### Removal of null results

**FIG. 4.**
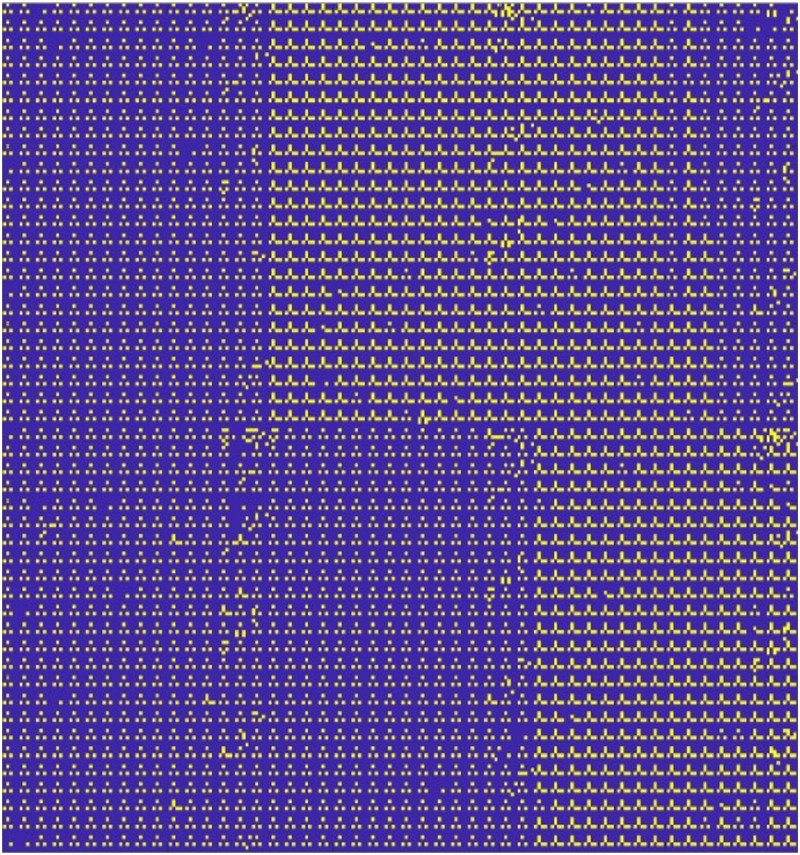
Detected blank dots in the array. Visible straight boundaries between regions of different blank dot densities derive from the technical feature of array production procedure and the absence of clones in the clonal library source plates.

The protein arrays contain null results that include marker dots and dots that do not produce any signal, hereby referred to as blank dots. Each cell can have up to two pairs of blank dots that should be excluded from subsequent statistical analyses. Marker dots were removed based on previously known positions, whereas blank dots were removed based on the divergence of the pixel intensity field. Dots with signals form sinks in the divergence field. Figure 4 shows the position of the blank dots and marker dots throughout the entire array.

### Background subtraction

We do not include any user input for background subtraction to improve reproducibility. To quantify the signal strength of each dot, the background pixel offset and gain should be subtracted. For large arrays with human-derived samples, local variations in offset and gain are significant. Variations were observed even within one cell. As such, we assume that offsets are only homogenous at the dot level. Within each dot, the background offset pixel value is defined as the intensity of the pixel(s) that expresses no luminance. As such, this corresponds to the pixel with minimum intensity. Dots with extremely strong signal can fill the entire dot space with luminant signal. In these cases, background pixel values would be abnormally high and detected as outliers. Outlier background values will be replaced by a local median background value instead.

**FIG. 5.**
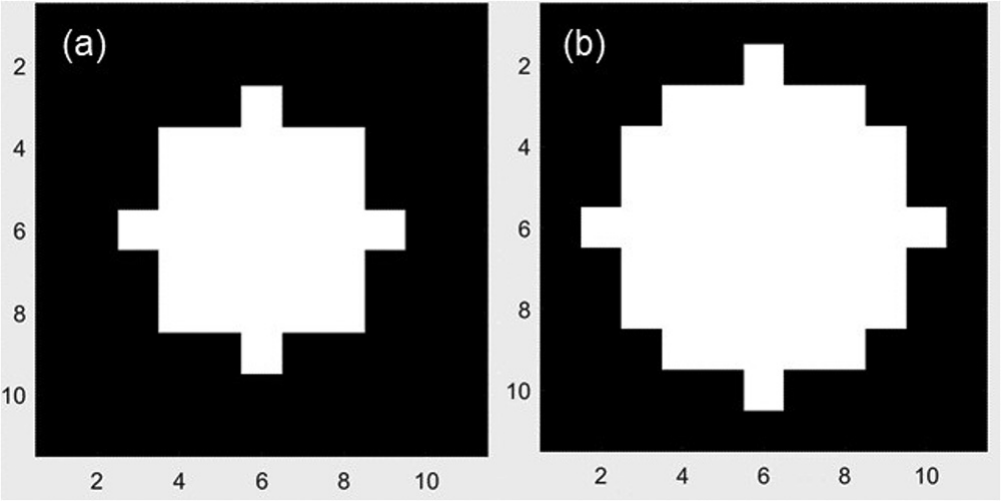
(a) Three-dot and (b) four-dot radius localities in an array. Numbers on the x and y axes of each radius locality area represent dot count coordinates.

In order to adjust for background, the specific locality (position) of the dot of interest within the parameters of the cell needs to be defined. The locality of a dot is, therefore, defined as the sample of dots contained within an area surrounding the dot of interest, similar to a moving window. To define the size of such an area, a specific radius of dots has to be defined taking into account competing factors. A small radius will have a higher homogeneity of pixel gain but suffers from statistically unreliable small samples. A three-dot radius completely covers the area of one cell with 29 dots. At the worst case, only four dots lie outside of the cell. As such, a three-dot radius locality can be susceptible to errors within a cell. A four-dot radius yields 49 dots, which includes 24 dots outside of the cell at worst. As such, a four-dot radius has been ultimately selected to define a dot locality for background analysis. Figure 5 compares a three-dot to a four-dot radius locality.

**FIG. 6.**
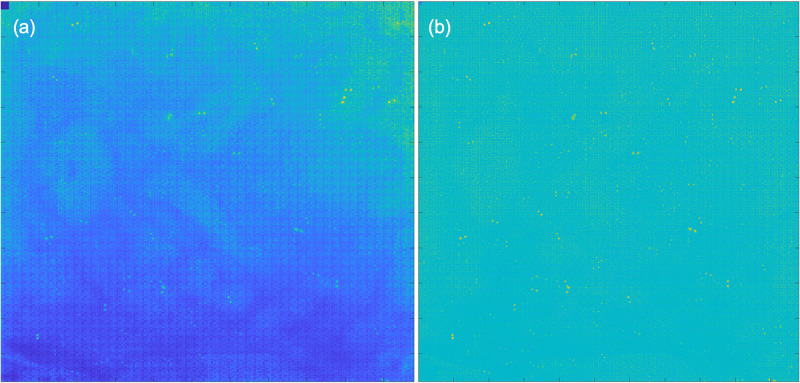
(a) Before and (b) after background subtraction. Note that our background subtraction method did not suppress the strong signals within the array.

Next, the entire array is reworked by subtracting each pixel with its corresponding offset (Fig. 6). Once the offset is removed, only the variable gain component remains. The gain component can be determined if a known reference value is available. Since the arrays do not contain a reference value, only relative gain values can be determined.

### Pixel value summation

Pixel values within each dot are summed to obtain the total pixel intensity. Unlike other platforms, we do not require a shape mask to the determine region of interest within the dot. In our case, the entire dot (9 × 9 pixels) will be included in the dot pixel sum since the background pixel value is negligible after the background subtraction process. The result is a 240 × 240 numerical array.

### Median absolute deviation (MAD) calculation

Since there is no reference to an absolute value, we define overexpression as a dot that has significantly higher intensity than dots in its locality. Since an overexpression is a statistical outlier, we measure the degree of spread of each dot from the local center of distribution in terms of pixel intensity. The median absolute deviation (MAD) is calculated for each locality. Each dot of interest will yield an absolute deviation from median, which is normalized with respect to its local MAD. As such, the local gain component is eliminated. The result is the number of MADs from median, which is reported as the relative quantification of expression levels. Since the local offset and gain components were removed, the number of MADs from median can be used to compare levels of expressions across arrays from different samples and time points.

**FIG. 7.**
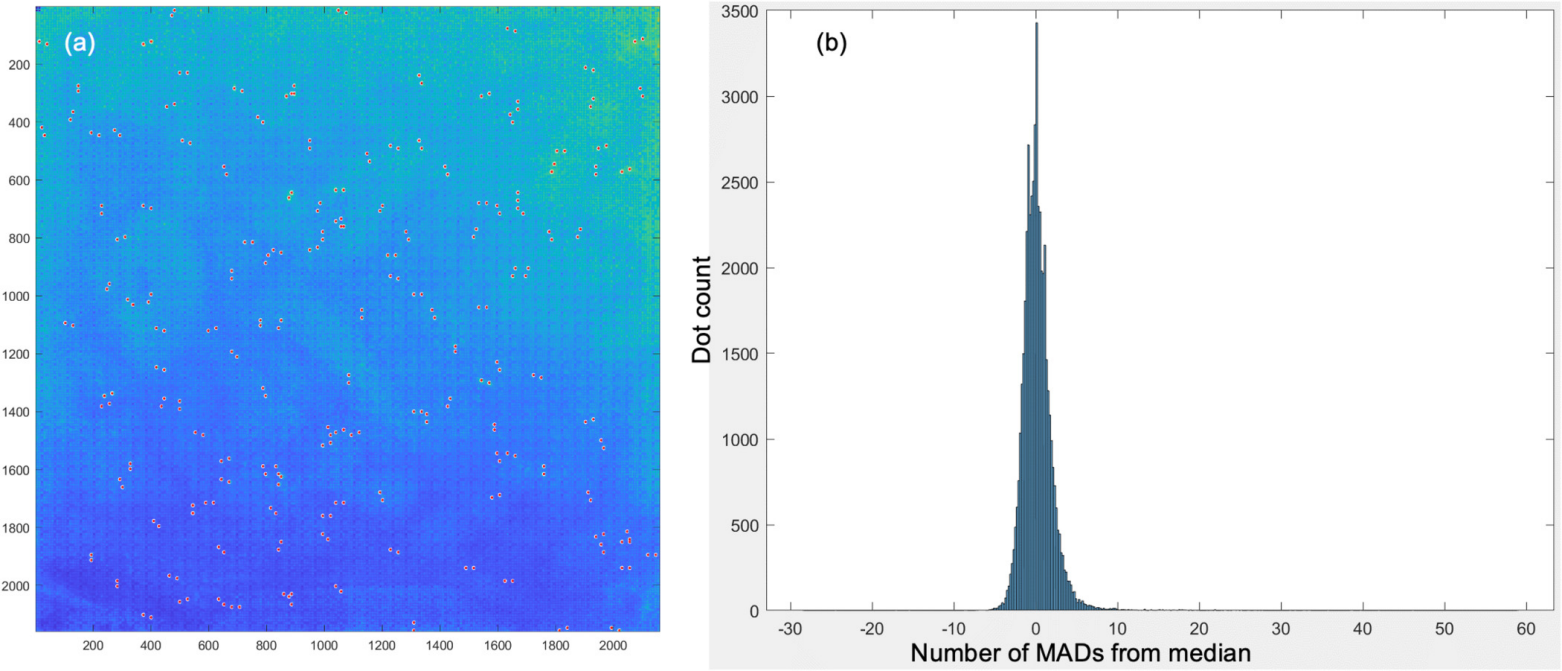
Output results. (a) Dot pairs with MAD > 3 highlighted in red. (b) Histogram of dot count vs number of MADs from median. Note that this array contains extremely strong signals that have MAD > 50.

The number of MADs from median for all 57 600 dots is stored in a linearized array with their corresponding x and y coordinates to facilitate matching with the protein library. Figure 7(a) illustrates the outliers detected based on the customizable MAD threshold > 3. It combines all procedures introduced throughout the manuscript depicting positive signals highlighted as red dot pairs. As the output is a generic numerical array, the results can be analyzed or filtered in Microsoft Excel or MATLAB. These results can be stacked for comparison or longitudinal study purposes.

## CONCLUSIONS

We developed a first, automated semiquantitative open-source software package for the analysis of widely used protein macroarrays. The software allows accurate single array and inter-array comparative studies through the tackling of intra-array inconsistencies arising from experimental disparities. The innovative and automated image analysis process includes adaptive positioning, background identification and subtraction, removal of null signals, robust statistical analysis, and protein pair validation. The normalized values allow a convenient semiquantitative data analysis of different samples or timepoints, enabling accurate characterization of sample series to identify relative changes for instance in clinical samples in response to diseases and treatment. The associated code is available for use in the supplementary information section.

## SUPPLEMENTARY MATERIAL

See the supplementary material for MATLAB code for protein array analysis.

## Data Availability

The data that support the findings of this study are available from the corresponding author upon reasonable request.
